# Pharmaceutical Wastage in Public Health Facilities of Ethiopia: Determinants, Consequences, and Solutions

**DOI:** 10.1002/puh2.70244

**Published:** 2026-04-27

**Authors:** Ewunetie Mekashaw Bayked, Adam Tesfaw Debeb, Getachew Moges, Husien Nurahmed Toleha, Teklehaimanot Fentie Wendie, Minimize Hassen

**Affiliations:** ^1^ Department of Pharmacy, College of Medicine and Health Sciences (CMHS) Wollo University Dessie Ethiopia; ^2^ Department of Pharmacy Asrade Zewudie Memorial Primary Hospital Burie Ethiopia

**Keywords:** consequences, determinants, Northwest Ethiopia, pharmaceutical wastage, public health facilities, solutions

## Abstract

**Background:**

Pharmaceutical wastage presents a significant challenge to health systems in developing countries, including Ethiopia; however, evidence regarding its magnitude and drivers remains limited. This study aimed to examine the extent of pharmaceutical wastage in public health facilities in Northwest Ethiopia, identify its key determinants and consequences, and explore feasible solutions to minimize losses and improve pharmaceutical efficiency. Unlike previous single‐method studies, this research integrates financial quantification with a qualitative exploration of systemic issues, thereby providing a comprehensive evidence base to inform policy and supply chain reforms.

**Methods:**

An explanatory‐sequential mixed‐methods design was employed in 17 public health facilities. A cross‐sectional survey of 121 health professionals, selected through stratified random sampling, quantified wastage and perceptions using self‐administered questionnaires. Descriptive statistics (MS Excel 2016, Statistical Package for the Social Sciences [SPSS] v26) were applied. In‐depth interviews with purposively selected key informants provided qualitative insights, thematically analyzed using QDA Miner Lite v2.0.9.

**Results:**

The total value of wasted pharmaceuticals was ETB 12,201,119.21 (approximately $235,089.00), calculated using the average 2022 exchange rate of $1 = 51.9 ETB, resulting in an overall wastage rate of 4.0%. A decline was observed from 4.72% in 2012 to 2.96% in the 2014 Ethiopian fiscal year. Expired products accounted for nearly 98% of the wastage value. Health professionals identified lack of accountability, receipt of near‐expiry products, and absence of inter‐facility transfer mechanisms as major causes. Key informants further highlighted facility, supplier, and system‐level weaknesses as contributing factors.

**Conclusion:**

Although wastage rates showed a declining trend, levels remain above the national standard, with expiration the leading cause. Strengthening accountability, introducing electronic inventory systems, and enabling redistribution of near‐expired products between facilities are essential to minimize losses and improve efficiency in resource‐limited health systems.

## Graphical abstract

A mixed‐methods study of 17 Ethiopian public health facilities found pharmaceutical wastage of ETB 12.2 million (4.0%), mainly from expiration (98%). Key drivers included weak accountability, near‐expiry deliveries, and poor forecasting. Strengthening electronic inventory systems and redistribution mechanisms can substantially reduce losses and improve health system efficiency.

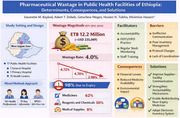

## 1 Introduction

One of the goals of the global development agenda and a crucial element of the right to health is having access to effective, affordable, and high‐quality medicines [1], because they are critical elements for the healthcare delivery system to prevent, diagnose, cure, mitigate, or treat disease [2]. They improve healthcare outcomes, customer satisfaction, and patient attendance when managed well [3]. If not, they become damaged, expired, obsolete, unsafe for use, and wasted [4]. Pharmaceutical wastage is the result of the accumulation of such unsafe pharmaceuticals [4]. It is unwanted pharmaceuticals that are no longer required and need to be disposed of appropriately [5]. This wastage reduces the availability of pharmaceuticals and the quality of healthcare. The supply cycle thus must be managed to prevent all types of wastage, such as pilferage, misuse, and expiration [6]. Poor stock rotation causes drugs to expire, which accounts for 4%–9% of total wastage [7].

Over 50% of all pharmaceuticals are prescribed, dispensed, or sold improperly worldwide, and 50% of patients do not take them as directed [8]. Pharmaceutical wastage is thus a global issue [9, 10, 11]. Almost 2 billion people worldwide lack access to essential medicines, which indicates that essential medicines are not available, affordable, accessible, acceptable, or of bad quality for more than a quarter of the world's population [12]. It is a major issue for healthcare delivery systems, even in developed countries. In Sweden and Austria, for instance, 2.3%–4.6% and 10%, respectively, of the volume dispensed were returned to pharmacies [9, 13]. Unused medications in the United States (US) are estimated to be between 3% and 7% [14].

Pharmaceutical wastage has a financial impact because it accounts for 25% of overall health spending, and 70% of cash invested in important drugs is squandered in standard supply systems [7]. For instance, in the United Kingdom (UK), wastage of National Health Service (NHS) prescribed medicines is estimated at £300 million annually [15]. The value of returned medicines in Vienna was €8.1 million [13]. Canada's wastage costs $8 billion annually [14].

Medication wastage has significant economic and environmental costs [5]. Unused or expired pharmaceuticals pose a health and environmental risk [16, 17]. Beyond economic loss, pharmaceutical wastage thus poses environmental and public health risks. Improper disposal of expired medicines can contaminate soil and water systems, contribute to antimicrobial resistance, and introduce hazardous chemical residues into ecosystems. These environmental externalities impose indirect economic costs on health systems and communities, further amplifying the importance of minimizing wastage [18].

Budget constraints and the wastage of unused pharmaceuticals in low‐ and middle‐income countries (LMICs) pose a significant threat to the healthcare system and economy [16, 17]. This exacerbates the burden on the already underfunded healthcare system in these nations [19]. Pharmaceutical wastage in developing countries diminishes the availability of essential medicines to patients and compromises the quality of care, leading to reduced access [7]. For example, in Rwanda, factors such as supply chain management, inadequate storage, and excess drug supply contribute to pharmaceutical expiry [20]. There are various issues with the Ethiopian pharmaceutical supply chain that require a systematic approach [9]. For instance, Ethiopia is largely reliant on the importation of medicines (80%) [21]. The most important challenges are pharmaceutical stock‐outs; wastage due to expiration, theft, and damage; a lack of transparent and accountable transactions and services; poor supplier performance; lengthy procurement lead times; poor record‐keeping and data quality; a lack of distribution vehicles for pharmaceuticals; insufficient follow‐up and support; a lack of performance monitoring and evaluation systems; training gaps; and a high staff turnover rate [9].

Pharmaceutical wastage is a major issue in this nation, with few studies highlighting its magnitude. The medicine wastage rate in the Southwest Shoa Zone of Oromia was 7.5% in 2017 [9]. The overall pharmaceutical wastage rate in Dessie town, Northeast Ethiopia, was 3.68% from 2015 to 2017 [22]. The wastage rate of reproductive health medicines in the West Wollega Zone of Ethiopia was 8.04% due to expiration [2]. A study conducted in the Dire Dawa City Administration identified several major factors contributing to pharmaceutical wastage: near‐expiration medicines, lack of clinician participation in medicine selection and quantification, abrupt changes in treatment practices, overstocked medicines due to improper forecasting, and a lack of accountability for stock‐outs and wastage [17].

The value of wastage in Ethiopia was estimated at 11,078,910.52 ETB, according to a study by regional health bureaus and the FMOH [9, 23]. The total financial loss from expired medicines in Gondar Town, Ethiopia, was $1337.60 [24]. In health facilities within the Arsi Zone, Oromia Regional State, Ethiopia, the total monetary value of expired medicines was $185,938.86. During the same period, these facilities received medicines valued at $2,425,882.64, resulting in an expiration rate of 7.66%. The leading classes of wasted medicines were anti‐infectives (35.51%) and central nervous system disorder medications (20.48%), primarily in solid dosage forms (48.81%), followed by liquid dosage forms (41.82%). Factors contributing to medicine expiration included the delivery of nearly expired medicines by the Ethiopian Pharmaceutical Supply Service (EPSS), the absence of an exchange system, and overstocked medicines resulting from improper forecasting [25].

In addition, another study conducted in Ethiopia qualitatively revealed that medicine wastage is a persistent problem in public health facilities due to poor communication, non‐compliance with the first‐expire‐first‐out principle, and weak monitoring systems [7]. Supplier‐related factors (pushing medicines in bulk without request) and demand‐related factors (less customer demand for some family planning methods) are contributing to drug expiration in the West Wollega zone of Ethiopia [2].

In general, prior studies identify recurrent drivers, including near‐expiry deliveries from suppliers, weak demand forecasting, inadequate stock rotation practices, limited accountability mechanisms, and insufficient inter‐facility redistribution systems [2, 7, 9, 17, 22, 23, 24, 25]. Despite these findings, most studies have focused on single facilities in specific regions and have relied predominantly on quantitative record reviews, limiting deeper exploration of systemic and managerial determinants. This study addresses the gap by integrating zonal‐level financial analysis with qualitative insights from key decision‐makers, offering a multidimensional perspective on pharmaceutical wastage. The objective was to determine the extent of pharmaceutical wastage and its contributing factors at public health facilities in the West Gojjam Zone, Ethiopia, using a mixed‐methods approach.

## 2 Methods

### 2.1 Study Setting

**FIGURE 1:**
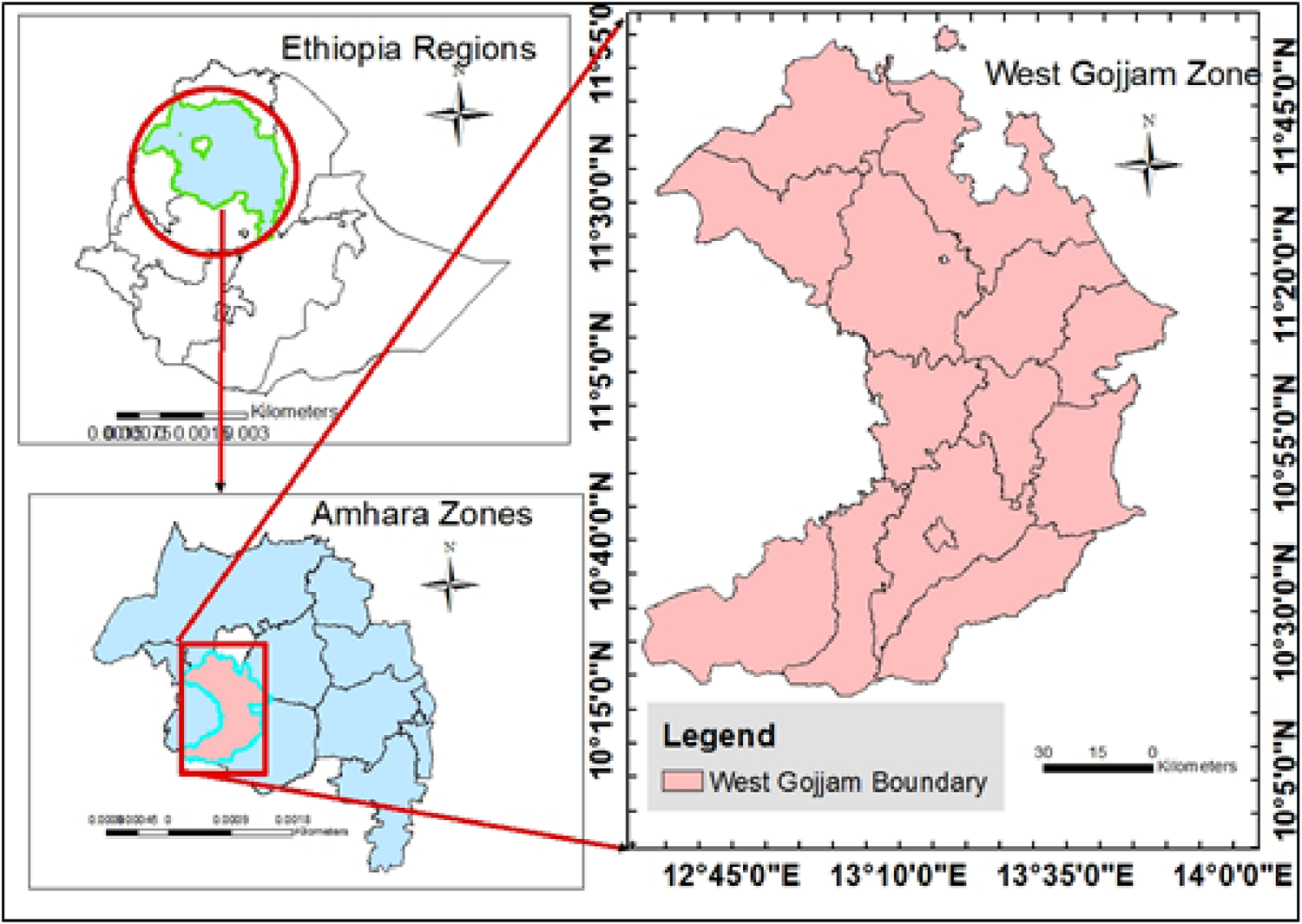
Map of the West Gojjam Zone, Ethiopia [28].

The study was conducted in the West Gojjam Zone (Figure 1), one of the zones in the Amhara regional state. Finoteselam, the zonal capital, is located 387 km from Addis Ababa, the capital of Ethiopia, and 176 km from Bahir Dar, the capital of the Amhara regional state [26]. In the Ethiopian fiscal year 2014, the zone had a total population of 2,795,775, comprising 1,395,091 men and 1,400,684 women [27]. According to a report by the Zonal Health Department, the zone has seven hospitals and 108 health centers.

### 2.2 Study Design and Period

From July 5 to August 30, 2022, we conducted explanatory‐sequential mixed‐methods research. This involved an institution‐based cross‐sectional study to assess the pharmaceutical wastage rate and perceived determinants in selected public health facilities, followed by a descriptive phenomenological investigation to deeply explore the underlying reasons for pharmaceutical wastage, its impact, and potential solutions for prevention or minimization.

### 2.3 Information Sources

All public health facilities in the West Gojjam Zone were considered the source population. This includes all health professionals in the zone involved in pharmaceutical supply chain management activities. Documents used for these activities, such as pharmaceutical wastage registration and disposal reports, bin cards, stock cards, the health commodity management information system, and Model 19, were also part of the source populations.

### 2.4 Study Population

The study examined health facilities in Ethiopia, focusing on health professionals responsible for managing the pharmaceutical supply chain. The assessment quantified pharmaceutical waste in the sampled facilities from fiscal years 2012 to 2014.

### 2.5 Sample Size Determination and Sampling Procedure

The number of necessary health facilities was determined using the Logistics Indicators Assessment Tool (LIAT) [29]. The document advises including at least 15% of the targeted health facilities in the survey. Using stratified sampling techniques, hospitals were first categorized into general and primary types, whereas health centers were divided into Type A and Type B on the basis of patient load and their status as anti‐retroviral therapy (ART) sites. Type A, characterized by a high patient load, serves as the distribution center for anti‐retroviral drugs. In contrast, Type B has a lower patient load and does not distribute anti‐retroviral therapy. Due to the presence of only one general hospital in the zone, it was purposely selected. Additionally, a proportionate number of primary hospitals and health centers from each level were chosen using a simple random sampling technique. Consequently, 1 general hospital, 1 primary hospital, 4 Type‐A health centers, and 11 Type‐B health centers were selected. These 17 health facilities were chosen from a pool of 115 health facilities that have been operational for more than 3 years.

For the quantitative aspect of the study, health professionals involved in the Drug and Therapeutics Committee (DTC) and pharmaceutical supply chain management activities were selected. These included roles such as pharmacy head, logistics officer, store manager, and DTC member. All DTC members and pharmacy/supply chain professionals working in the selected 17 public health facilities during the study period were targeted. Therefore, the quantitative survey constituted a census of eligible professionals rather than a sampled subset. Where non‐response occurred, replacement was not applied.

For the qualitative study, key informants were purposefully selected, including chief executive officers (CEOs) of health facilities, heads of pharmacy departments, and storemen from chosen public health facilities. These individuals were considered to possess more extensive information than other health professionals. The sample size for the qualitative study was determined by the saturation point of information provided by these key informants. Saturation was observed when the last four consecutive key informants expressed similar ideas, at which point the in‐depth interviews were terminated [30]. In total, 15 key informants were interviewed.

### 2.6 Inclusion and Exclusion Criteria

The eligibility criteria included public health facilities that had been operational for more than 3 years; staff involved in DTC and pharmaceutical management activities who were available and volunteered during the study period; and pharmaceuticals recorded as wasted, with prices noted between the 2012 and 2014 fiscal years. The inclusion criteria included pharmaceuticals classified as free, which were sourced from programs and donations, as well as health posts situated in the West Gojjam Zone.

### 2.7 Data Collection Instrument and Techniques

#### 2.7.1 Data Collection Instrument

Quantitative data were collected using a sheet adapted from the LIAT [29] to determine pharmaceutical wastage value. For pharmaceutical wastage, the data collection sheet was prepared in accordance with the Ethiopian Food and Drug Authority's (EFDA's) pharmaceutical waste management and disposal directive [31]. A self‐administered questionnaire, which included questions about sociodemographic characteristics (age, sex, work experience, and education level) and perceived factors contributing to pharmaceutical wastage, was also used [6, 7]. Questions from the data collection tools that were not relevant to the study were omitted, and modifications were made to the language of some questions for easier comprehension by the participants. Each item was evaluated on a 5‐point Likert scale, ranging from 1 (strongly disagree) to 5 (strongly agree).

For qualitative data, a semi‐structured interview guide was prepared on the basis of the analysis of quantitative findings. This guide aimed to explore key informants’ perspectives on contributing factors, consequences, and potential solutions to mitigate the issue at hand. The preparation of this interview guide was informed by a comprehensive literature review.

#### 2.7.2 Data Collection Techniques

Two pharmacists collected quantitative data using data abstraction formats and self‐administered questionnaires. To estimate the value of pharmaceutical waste and the wastage rate, retrospective data extraction was used to record pharmaceuticals marked as expired in each health facility studied. Records of pharmaceutical waste, disposal reports, bin cards, stock cards, the health commodity management information system, and Model 19 were reviewed to abstract secondary data. The values of wasted pharmaceuticals were calculated on the basis of their costs, as recorded in the pharmaceutical wastage registration and disposal lists. The total value of received or purchased pharmaceuticals was determined using the price recorded on Model 19.

The second author (A.T.) collected qualitative data through in‐depth interviews with the CEOs, pharmacy case team leaders, and store managers. A voice recorder was used to document the interviews, with key points noted down separately. Each interview lasted approximately 38 min and took place in a private setting. The interviews were conducted every other day to provide sufficient time for the transcription, translation, and coding of each session.

### 2.8 Data Management and Analysis

All collected data underwent a thorough examination for completeness, accuracy, clarity, and consistency during the collection process. Responses to each question were individually coded. The quantitative data were inputted into MS Excel 2016 and the Statistical Package for the Social Sciences (SPSS) version 26 for encoding and analysis. SPSS was selected for its reliability in handling structured survey data and financial datasets, facilitating accurate ranking and comparative analysis [32]. The analysis was performed collectively and included descriptive statistics such as frequency, mean, standard deviation (SD), percentages, and charts. The Likert‐scale items assessing perceived causes of pharmaceutical wastage were analyzed using mean scores and SDs. Items were ranked on the basis of their mean values to identify the most strongly perceived contributing factors. In the qualitative portion of the study, interviews were initially recorded and later transcribed verbatim into MS Word after multiple listenings.

The pharmaceutical wastage rate is a percentage derived from dividing the monetary value of wasted pharmaceuticals by the total value available for sale. This total value is calculated by adding the initial value of pharmaceuticals in the budget year to the value of pharmaceuticals received during that same year, as demonstrated in the following equation [33]:

$$\begin{array}{ccc} & & \mathrm{Pharmaceutical}\;\mathrm{waste}\;\mathrm{rate}\;\left(\%\right)\\ & & \;=\frac{\mathrm{Value}\;\mathrm{of}\;\mathrm{wasted}\;\mathrm{pharmaceuticals}\;\mathrm{in}\;a\;\mathrm{budget}\;\mathrm{year}}{\mathrm{The}\;\mathrm{total}\;\mathrm{value}\;\mathrm{available}\;\mathrm{for}\;\mathrm{sale}\;\mathrm{during}\;\mathrm{that}\;\mathrm{period}}\times 100 \end{array}$$

For the qualitative approach, the transcripts were thoroughly reviewed before being translated into English to ensure a comprehensive understanding of the data. Subsequently, open coding was used to identify emerging themes through thematic analysis [30, 34]. The QDA Miner Lite V3 software was utilized for the organization, sorting, and manipulation of materials to streamline the analytical process.

Preliminary quantitative findings, particularly the high proportion of expiry‐related wastage and the ranking of perceived contributing factors such as near‐expiry deliveries and lack of accountability, were used to inform the development of the qualitative interview guide. Specific probes were constructed to explore these issues in greater depth during the in‐depth interviews, thereby strengthening the explanatory‐sequential design.

The monetary value of wasted pharmaceuticals was calculated using documented wastage reports and stock records obtained from each facility. Unit costs were extracted from procurement records, and total wastage values were computed by multiplying quantities by corresponding unit prices. Interview data were used to contextualize underlying causes but did not determine financial calculations.

### 2.9 Data Quality Assurance

The questionnaire for the quantitative study was pre‐tested at two health centers (one Type A and one Type B), which were not part of the study, to verify the tool's practicality. Data collectors, who were pharmacy professionals (pharmacists), received a day's training on the data collection instruments and procedures prior to the actual collection. The principal investigator supervised the data collection process, promptly addressing any inconsistencies. The interview guide for the in‐depth interview was meticulously reviewed by all authors. Initially prepared in English, the guide was translated into Amharic and then retranslated back into English to ensure message consistency. The Amharic version was used for interviews with key informants.

### 2.10 Reflexivity

As an insider, the qualitative data collector (ATD) gained significant insights into the professional challenges of the study. He contemplated how the data collection process shaped his perspective and influenced the responses of other participants. Being perceived as a senior pharmacy professional and an elite member posed certain navigational challenges. To mitigate these, he employed open‐ended questions and facilitated informal discussions on the topics introduced. Furthermore, he maintained impartiality by setting aside his pre‐existing or theoretical knowledge of pharmaceutical wastage and its contributing factors during the in‐depth interview process and data analysis.

## 3 Results

Seventeen public health facilities, comprising one general hospital, one primary hospital, and 15 health centers, were surveyed. The facilities had an average operational duration of 22.41 years (SD = 12.94). The youngest facility was 4 years old, whereas the oldest had been operational for 60 years, as illustrated in Table 1.

**TABLE 1:** Characteristics of the health facilities surveyed in West Gojjam Zone, Ethiopia (*n* = 17), 2022.

Facility code	Type	Level	Establishment
FSGHOS	Hospital	General hospital	1954
ASZMPHOS	Hospital	Primary hospital	2008
GISHAHC	Health center	Type‐A HC	1988
ALEHC	Health center	Type‐B HC	1998
SHIHC	Health center	Type‐A HC	1987
BIRHC	Health center	Type‐B HC	2002
DENHC	Health center	Type‐A HC	1987
TIAHC	Health center	Type‐B HC	2003
MANHC	Health center	Type‐B HC	1992
GIGHC	Health center	Type‐B HC	1992
WOGHC	Health center	Type‐B HC	1991
AGOHC	health center	Type‐B HC	2010
AGUHC	Health center	Type‐B HC	1992
WAHC	Health center	Type‐B HC	1995
KUHC	Health center	Type‐B HC	1991
BURHC	Health center	Type‐A HC	1974
YECHC	Health center	Type‐B HC	1993

### 3.1 Quantitative Findings

#### 3.1.1 The Extent of Pharmaceuticals’ Wastage Rate

The surveyed health facilities wasted pharmaceuticals with a total monetary value of 12,201,119.20 ETB from EFY 2012–2014. During the same period, these facilities had pharmaceuticals worth 304,404,339.90 ETB available for sale. This resulted in a wastage rate of 4.00%. In the EFY 2012, there was a total wastage of 3,945,246.86 ETB. During this same period, all surveyed health facilities had pharmaceuticals available for sale worth 83,461,047.19 ETB, representing an average wastage rate of 4.72%. In EFY 2013, the estimated wastage value increased to 4,612,939.81 ETB. Concurrently, the value of pharmaceuticals available for sale in all surveyed health facilities was 97,983,568.84 ETB, resulting in an annual wastage rate of 4.70%. In the 2014 fiscal year, an overall wastage of 3,642,932.54 ETB was recorded.

During the same period, all surveyed health facilities offered pharmaceuticals for sale worth 122,959,723.97 ETB, resulting in an estimated annual wastage rate of 2.96%. However, if the results from the hospitals were excluded from this analysis, the average wastage rate in the remaining health centers would increase to 4.98%, amounting to a loss of 7,252,428.79 ETB. In the two hospitals surveyed, the total pharmaceutical wastage rate was estimated at 3.11%, amounting to a value of 4,948,690.42 ETB. In Type “A” health centers, the total pharmaceutical wastage rate was estimated at 4.45%, with a value of 2,310,086.85 ETB. Meanwhile, in Type “B” health centers, the total pharmaceutical wastage rate was estimated at 5.27%, equating to a value of 4,942,341.94 ETB, as illustrated in Table 2.

**TABLE 2:** The value of pharmaceuticals (available for sale and wasted) and estimation of the total pharmaceutical waste rate in the facilities (2012–2014 EFY), West Gojjam, Ethiopia.

	2012			2013			2014		
Health facility code	Available for sale in ETB	Wasted in ETB	%	Available for sale in ETB	Wasted in ETB	%	Available for sale in ETB	Wasted in ETB	%
FSGHOS	29,178,122.5	847,306.0	2.9	35,604,587.3	2,020,508.0	5.6	47,070,521.0	948,018.0	2.0
ASZMPHOS	11,364,728.3	384,217.7	3.3	13,057,496.3	353,154.5	2.7	22,563,763.4	395,486.1	1.7
GISHAHC	2,945,943.7	175,473.4	5.9	3,406,163.4	192,946.5	5.6	3,723,415.6	209,318.9	5.6
ALEHC	2,945,541.5	150,346.8	5.1	2,763,237.4	102,453.6	3.7	2,973,474.8	107,423.7	3.6
SHIHC	2,963,642.6	151,446.7	5.1	2,764,437.3	102,745.6	3.7	2,983,784.6	107,645.8	3.6
BIRHC	2,712,671.3	213,136.4	7.8	2,741,643.6	153,903.8	5.6	3,186,435.7	208,827.70	6.5
DENHC	4,976,433.0	208,231.7	4.1	4,500,079.8	160,165.0	3.5	3,705,473.6	108,302.7	2.9
TIAHC	1,845,540.6	89,315.9	4.8	2,228,236.4	82,426.3	3.6	2,558,579.9	105,423.6	4.1
MANHC	1,945,941.7	192,246.3	9.8	3,481,712.2	184,343.7	5.2	3,723,025.7	209,217.9	5.6
GIGHC	1,976,999.7	193,346.4	9.7	3,997,813.2	187,446.8	4.6	3,823,275.7	209,643.7	5.4
WOGHC	2,768,457.0	132,679.8	4.7	2,397,926.9	92,165.3	3.8	3,256,473.6	101,343.7	3.1
AGOHC	1,697,932.1	47,901.8	2.8	1,506,907.8	37,495.8	2.4	2,992,522.2	61,963.8	2.0
AGUHC	2,145,841.8	197,263.4	9.1	2,981,723.7	163,346.6	5.4	3,763,126.3	208,463.1	5.5
WAHC	2,813,718.4	215,236.5	7.6	3,163,463.6	184,896.7	5.8	2,987,674.8	193,837.7	6.4
KUHC	2,945,971.7	216,246.3	7.3	3,483,712.4	187,376.7	5.3	3,786,635.7	209,927.9	5.5
BURHC	5,797,103.5	421,273.3	7.2	7,488,484.1	315,392.9	4.2	6,548,067.5	157,144.2	2.4
YECHC	2,436,457.7	109,578.4	4.5	2,415,943.4	92,171.7	3.8	3,313,473.7	100,943.7	3.0
**Average**	**83,461,047.19**	**3,945,246.86**	**4.72**	**97,983,568.84**	**4,612,939.81**	**4.70**	**122,959,723.97**	**3,642,932.54**	**2.96**

*Note:* Available for sale = beginning stock + received stock.

##### 3.1.1.1 The Categories of Expired Pharmaceuticals

In total, 385 kinds of wasted pharmaceuticals were documented across all health facilities from 2012 to 2014 EFY. The expired pharmaceuticals were categorized by value as follows: program medicines (39.99%), budget medicines (22.01%), program reagents and chemicals (18.90%), budget reagents and chemicals (11.10%), program medical supplies (4.16%), and budget medical supplies (3.84%). Table 3 illustrates the estimated value of expired pharmaceuticals by category, indicating that the estimated value of expired medicines was the highest at 62%, followed by reagents and chemicals at 30% and medical supplies at 8%.

**TABLE 3:** Estimated value of expired pharmaceuticals by category in the study facilities (2012–2014 EFY), West Gojjam Zone, Ethiopia.

Category of expired pharmaceuticals		Value of expired pharmaceuticals, ETB (%)
**Medicines**	Program medicines	4,879,227.568 (39.99)
	Budget medicines	2,685,466.336 (22.01)
**Reagents and chemicals**	Program reagents and chemicals	2,306,011.5288 (18.90)
	Budget reagents and chemicals	1,354,324.2312 (11.10)
**Medical supplies**	Program medical supplies	507,566.559 (4.16)
	Budget medical supplies	468,522.977 (3.84)

#### 3.1.2 Trend of Pharmaceuticals’ Wastage Rate

**FIGURE 2:**
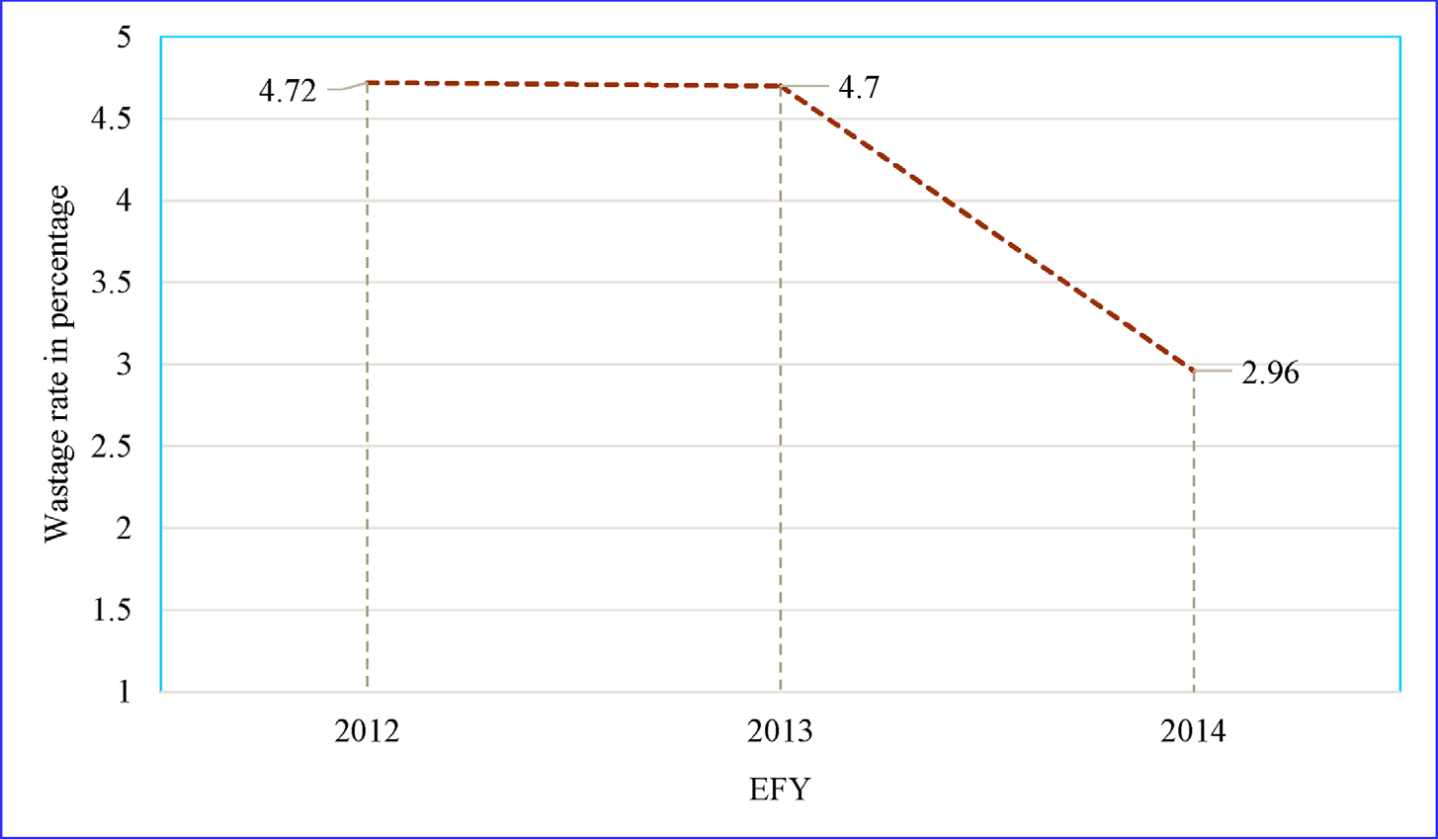
Average pharmaceuticals’ wastage rate of all sampled public health facilities in West Gojjam Zone, Ethiopia (2012–2014 EFY), 2022.

All selected public health facilities experienced a general reduction in the average wastage rate. The wastage rate of pharmaceuticals decreased from 4.72% in EFY 2012 to 2.96% in EFY 2014 across all health facilities sampled (refer to Figure 2).

#### 3.1.3 Reasons for Pharmaceuticals’ Wastage

**FIGURE 3:**
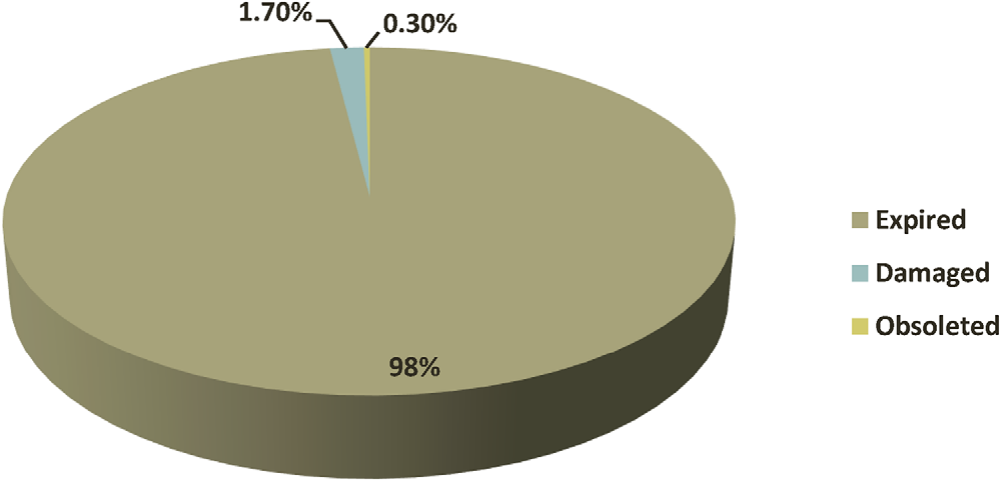
Reasons for wastage of pharmaceuticals in the sampled public health facilities, West Gojjam Zone, Ethiopia (2012–2014 EFY), 2022.

Pharmaceutical waste was primarily due to expiry, damage, and obsolescence (products removed from the market before their expiry date). The current study revealed that approximately 98% of the total value of wasted pharmaceuticals resulted from expiration, costing 298,316,253 ETB between EFY 2012 and 2014. During the same period, the total costs of damaged or deteriorated pharmaceuticals and obsolete pharmaceuticals were 5,174,873.80 ETB and 913,213.02 ETB, respectively. Notably, none of the health facilities sampled documented theft and pilferage as a cause of pharmaceutical wastage, as depicted in Figure 3.

#### 3.1.4 Factors Contributing to Pharmaceutical Wastage as Perceived by Health Professionals

A total of 125 questionnaires were distributed to health professionals involved in DTC and other pharmaceutical management activities within the studied health facilities. Of these, 121 were completed and returned, yielding a response rate of 96.8%. The survey included 67 respondents aged 20–30 years and 54 respondents aged 31–40 years. The age range of the participants was 25–37 years. Regarding their field of study, 65 (53.7%) were pharmacy professionals, including pharmacists and druggists, whereas 56 (46.3%) were medical practitioners such as doctors, health officers, nurses, and laboratory technicians. A majority of the respondents, 83 (68.6%), had 5–10 years of work experience (refer to Table 4).

**TABLE 4:** Sociodemographic characteristics of health professionals working in the selected public health facilities in West Gojjam Zone (*n* = 121), 2022.

Sociodemographic profile		Number	Percentage
Sex	Male	98	81.0
	Female	23	19.0
Age	20–30 years	67	55.4
	31–40 years	54	44.6
Profession	Medical doctor	8	6.6
	Pharmacy	65	53.7
	Nurse	18	14.9
	Health officer	18	14.9
	Laboratory	10	8.3
	Midwife	2	1.6
Level of education	Diploma	49	40.5
	Degree	55	45.5
	Master	17	14.0
Work experience	<5 years	23	19.0
	5–10 years	83	68.6
	>10 years	15	12.4

Health professionals engaged in DTC and pharmaceutical supply management were surveyed to evaluate their perceptions of the determinants of pharmaceutical wastage within their facilities, using a 5‐point Likert scale. The factors with the highest mean scores—reflecting those most strongly perceived as drivers of wastage—were lack of accountability for stock‐outs and wastage at the facility level (3.81); receipt of near‐expiry pharmaceuticals (less than 6 months remaining) (3.80); absence of a formal mechanism to redistribute nearly expired pharmaceuticals between facilities (3.67); lack of electronic inventory management systems capable of automatically capturing wastage data (3.65); inadequate communication and coordination with key stakeholders, including the health bureau/office, suppliers, and NGOs (3.55); and overstocking attributable to inaccurate demand forecasting within the facility (3.51) (Table 5).

**TABLE 5:** The perceived factors contributing to pharmaceutical wastage by health professionals in selected public health facilities, West Gojjam Zone, Ethiopia (*n* = 121).

Contributing factors	1 no. (%)	2 no. (%)	3 no. (%)	4 no. (%)	5 no. (%)	Mean
Lack of accountability for stock‐outs and wastage	7 (5.8)	12 (9.9)	8 (6.6)	63 (52.1)	31 (25.6)	3.8182
Delivery of nearly expired pharmaceuticals	10 (8.3)	12 (9.9)	8 (6.6)	53 (43.8)	38 (31.4)	3.8017
Lack of a system to move nearly expired pharmaceuticals	12 (9.9)	12 (9.9)	5 (4.1)	67 (55.4)	25 (20.7)	3.6694
Lack of electronic stock management tools	27 (22.3)	4 (3.3)	0 (0)	75 (62.0)	15 (12.4)	3.6446
Poor communication and coordination between stakeholder	5 (4.1)	19 (15.7)	9 (7.4)	81 (66.9)	7 (5.8)	3.5455
Overstock due to improper forecasting of needs	2 (1.7)	30 (24.8)	0 (0)	82 (67.8)	7 (5.8)	3.5124
Abrupt changes in treatment practices	8 (6.6	14 (11.6)	14 (11.6)	78 (64.5)	7 (5.8)	3.5124
Poor stock management, not using FEFO technique	14 (11.6)	16 (13.2)	11 (9.1)	55 (45.5)	25 (20.7)	3.5041
Weak or no mechanisms to manage pharmaceutical waste	4 (3.3)	36 (29.8)	7 (5.8)	63 (52.1)	11 (9.1)	3.3388
The absence of functional DTC in the health facilities	26 (21.5)	8 (6.6)	15 (12.4)	50 (41.3)	22 (18.2)	3.2810
Lack of accurate data for pharmaceutical quantification	19 (15.7)	17 (14.0)	7 (5.8)	71 (58.7)	7 (5.8)	3.2479
Selection of pharmaceuticals without essential medicines list	18 (14.9)	29 (24.0)	2 (1.7)	59 (48.8)	13 (10.70	3.1653
Clinicians non‐participation in selection and quantification	15 (12.4)	31 (25.6)	22 (18.2)	40 (33.1)	13 (10.7)	3.0413
Salaries of staff that are significantly lower for self‐support	2 (1.7)	55 (45.5)	9 (7.4)	49 (40.5)	6 (5.0)	3.0165
The shortage of pharmacy human resources in the facility	15 (12.4)	42 (34.7)	16 (13.6)	37 (30.6)	11 (9.1)	2.8926
Purchasing pharmaceuticals without plan/policy of facilities	26 (21.5)	26 (21.5)	18 (14.9)	41 (33.9)	10 (8.3)	2.8595
Limited knowledge and skills in drug supply management	20 (16.5)	46 (38.0)	11 (9.1)	32 (26.4)	12 (9.9)	2.7521
Weak physical security during the transportation	16 (13.2)	59 (48.8)	16 (13.2)	17 (14.0)	13 (10.7)	2.6033
Storage on the floor or not arranged systematically on shelves	25 (20.7)	42 (34.7)	23 (19.0)	25 (29.7)	6 (5.0)	2.5455
Failure to store heat‐sensitive items in functional refrigerators	32 (26.4)	44 (36.4)	11 (9.1)	21 (17.4)	13 (10.7)	2.5289

*Note:* 1 = strongly disagree, 2 = disagree, 3 = neutral, 4 = agree, and 5 = strongly agree.

Abbreviation: DTC, Drug and Therapeutics Committee.

### 3.2 Qualitative Findings

The quantitative survey identified lack of accountability and near‐expiry deliveries as the highest ranked perceived contributors to pharmaceutical wastage. These findings were further explored during the qualitative phase, where key informants elaborated on the systemic and operational dimensions underlying these factors.

#### 3.2.1 Sociodemographic Descriptions

A descriptive phenomenological study, utilizing in‐depth interviews, identified three patterns of responses: determinants of pharmaceutical wastage, the impact of pharmaceutical wastage on service provision, and suggestions for reducing pharmaceutical wastage. In‐depth interviews were conducted with 15 participants, comprising CEOs of health facilities, pharmacy case team heads, and store managers. All but two key informants were male. Seven participants held an MSc, whereas the remainder held a BA or BSc. Participant ages ranged from 25 to 36 years (29.3 ± 3.3). Participants’ work experience ranged from 5 to 13 years (*M* = 7.9, SD = 2.7) and included roles such as CEOs, pharmacy heads, and store managers (see Table 6).

**TABLE 6:** Sociodemographic descriptions of the key informants (*n* = 15), 2023.

Respondents	Sex	Age (year)	Education level	Experience (year)	Current position
P1	Male	34	MSc	10	Hospital CEO
P2	Male	32	MSc	9	Pharmacy head
P3	Male	36	MSc	13	Hospital CEO
P4	Male	28	MSc	7	Pharmacy head
P5	Male	31	MSc	11	Health center CEO
P6	Male	29	BSc	7	Pharmacy head
P7	Male	30	BSc	9	Health center CEO
P8	Female	28	BSc	5	Pharmacy head
P9	Male	32	MSc	9	Health center CEO
P10	Male	27	BSc	5	Pharmacy head
P11	Male	31	MSc	12	Health center CEO
P12	Female	26	BSc	6	Pharmacy head
P13	Male	25	BSc	5	Store manager
P14	Male	25	BSc	6	Pharmacy head
P15	Male	26	BSc	5	Store manager

Abbreviation: CEO, chief executive officer.

#### 3.2.2 Thematic Results

The thematic analysis identified three overarching themes: facilitators, consequences, and perceived solutions to pharmaceutical wastage. These themes were further organized into nine subthemes, capturing the diverse patterns and nuances of participants’ responses.

##### 3.2.2.1 Facilitators (Reasons) for Pharmaceutical Wastage

**FIGURE 4:**
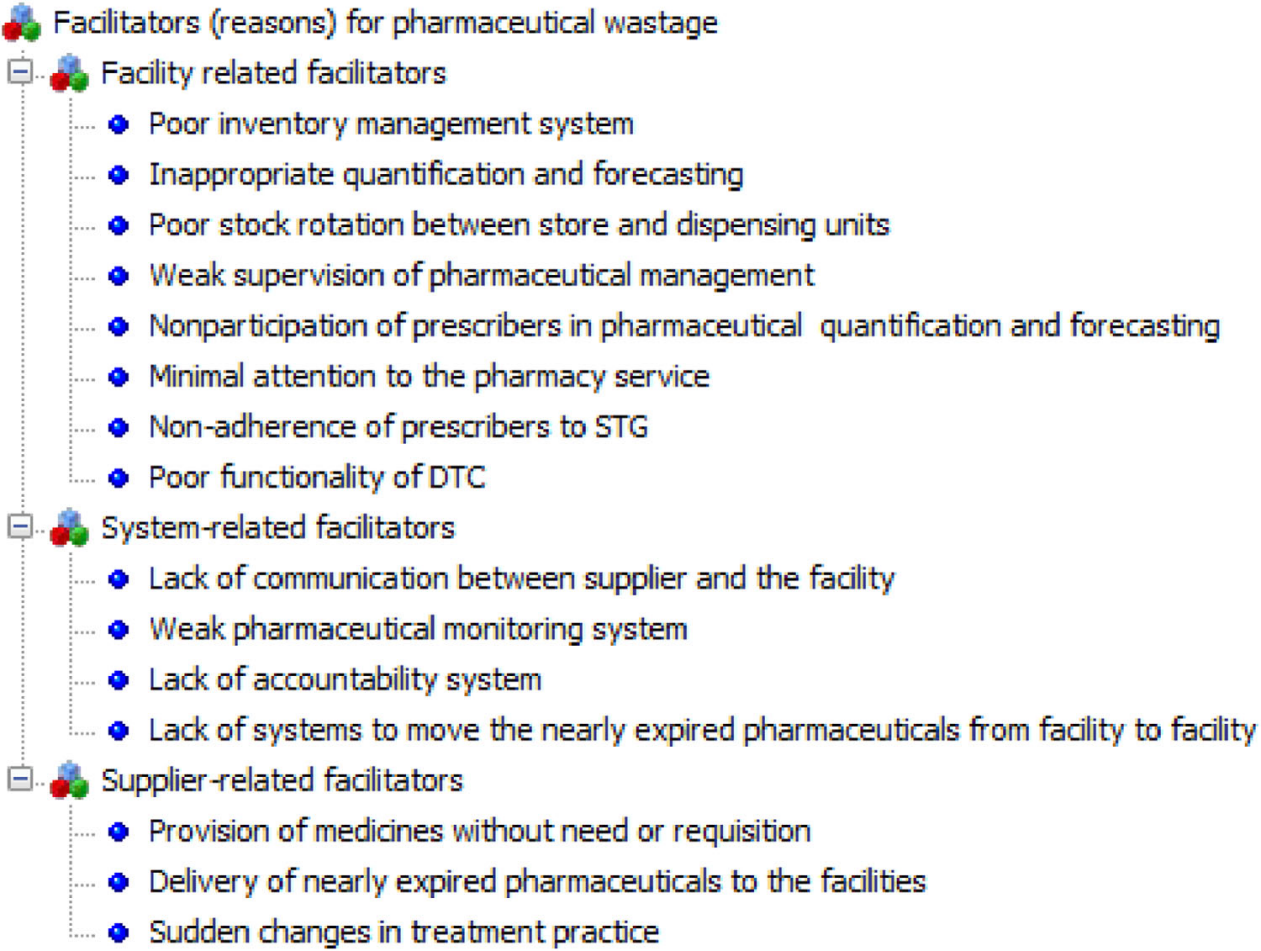
Perceived facilitators or reasons to pharmaceutical wastage, 2023. DTC, Drug and Therapeutics Committee.

The key informants identified several reasons contributing to pharmaceutical wastage in their facilities. These reasons were categorized into three themes: supplier‐related, facility‐related, and system‐related (Figure 4). Supplier‐related factors included the provision of pharmaceuticals without need or requisition, the delivery of nearly expired pharmaceuticals to the health facility, and sudden changes in treatment practices. These reasons were highlighted by statements made by the key informants:

*The EPSS and other partners frequently deliver large quantities of pharmaceuticals, particularly program‐specific medications with short expiration dates. A significant portion of these pharmaceuticals remains unused and subsequently expires in bulk at our facility. (P14)*

*The EPSS sometimes provides pharmaceuticals that have a short expiration date. If you refuse to receive these pharmaceuticals, they will not give you the other required pharmaceuticals listed on the same voucher. So, we were forced to receive the pharmaceuticals, knowing that they would expire before they were consumed. (P2)*

*Sometimes, program pharmaceuticals are missing for a long time. When they arrive, the EPSS gives them to us in bulk, which leads to their expiration. (P4)*

*Several factors contribute to pharmaceutical wastage. The primary contributors to this issue are the delivery of near‐expiry pharmaceuticals to facilities, particularly program‐specific medicines, and sudden changes in treatment practices. In our health facility, program medicines such as ART and tuberculosis medications are especially prone to wastage due to frequent modifications in treatment protocols. (P8)*

With respect to facility‐level challenges, key informants identified several critical shortcomings in stock management practices. These included failure to apply first‐in, first‐out (FIFO) or FEFO principles; quantification of pharmaceuticals based on imprecise or incomplete data; absence of an electronic stock management system; limited involvement of prescribers in quantification and forecasting processes; lack of a functional DTC; and weak communication between the EPSS and health facilities. These concerns were underscored in the following statement from a key informant:

*I think there are several reasons for the pharmaceuticals' wastage; for me, the presence of weak stock management and a lack of appropriate quantification and forecasting were the major issues. For example, if we do not use the FIFO or FEFO system, pharmaceutical wastage may increase, and when we procure pharmaceuticals, most of the time we consider the absence of stock rather than the accurate consumption data, which leads to overstock and expiry. (P1)*

Two key informants mentioned that the poor attitude of health facility administrators toward the pharmacy service and the lack of regular discussion with key stakeholder groups on issues contribute to pharmaceuticals’ wastage. One key informant said:

*In my view, health facility administrators often demonstrate a negative attitude toward pharmacy services. This is particularly evident in health centers, where proposals or initiatives put forward by pharmacy staff frequently fail to receive managerial support. In many cases, we are not even given the opportunity to raise concerns related to pharmaceutical supply. I believe this lack of support and open communication significantly contributes to pharmaceutical wastage. (P10)*

Key informants identified several system‐related issues, including a lack of accountability, poor communication between suppliers and facilities, a weak pharmaceutical monitoring system, and the absence of a system for transferring nearly expired pharmaceuticals between facilities. One key informant illustrated this by stating:

*I think multiple factors could be contributing to pharmaceutical wastage. However, the lack of a system of accountability in the facility was the major factor. (P13)*

Another one added to this:

*I don't think a single factor contributes to pharmaceutical waste in our facility. It's a result of various internal and external factors, but the lack of a system to move nearly expired pharmaceuticals between facilities is a major one. If we had a strong system to move near‐expired and overstocked items to other facilities with low stock, we would reduce pharmaceutical waste and even improve effective budget consumption. (P12)*

##### 3.2.2.2 Consequences of Pharmaceutical Wastage

**FIGURE 5:**
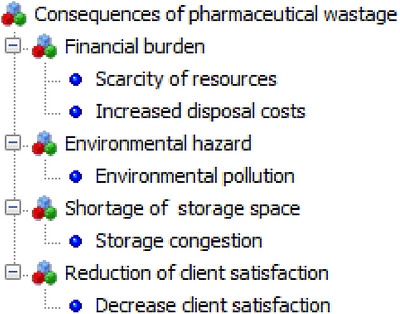
Perceived consequences of pharmaceutical wastage, 2023.

Key informants were asked for their opinion on how pharmaceutical wastage affected their service provision. They mentioned several consequences, including financial burdens (budget constraints), environmental pollution, a shortage of storage space, and reduced client satisfaction (Figure 5). All key informants stated that the biggest challenge related to pharmaceutical wastage was congested storage. According to them, a large portion of the pharmaceutical store's space is taken up by wasted pharmaceuticals. For example, one respondent said:

*Expired and damaged pharmaceuticals take up a lot of storage space, which means there's less room for usable ones. This then leads to a drop in quality, and customers can't get the effective, high‐quality medications they need. (P3)*

Another one added to this:

*Pharmaceutical waste has far‐reaching consequences, affecting both areas within and beyond the health facility. It poses environmental risks and places additional strain on healthcare financing and limited storage space budgets. Consequently, we consistently prioritize community and environmental considerations when managing and disposing of pharmaceutical waste. (P5)*

##### 3.2.2.3 Perceived Solutions to Minimize Pharmaceutical Wastage

**FIGURE 6:**
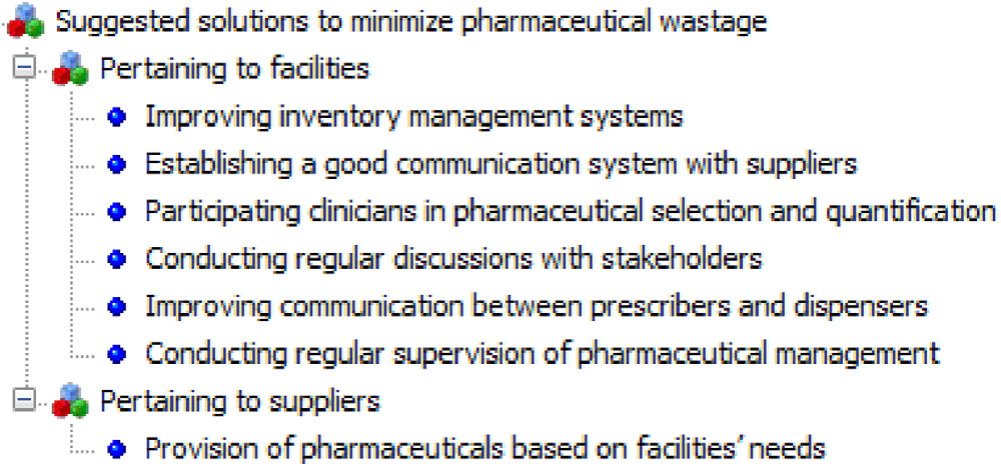
Perceived solutions to pharmaceutical wastage, 2023.

The respondents were invited to provide ideas on how to reduce pharmaceutical wastage in public health facilities. Their opinions were then grouped into two major categories: supplier‐related and facility‐related (Figure 6).

Facility‐related suggestions include improving inventory management systems, establishing a good communication system with suppliers, involving clinicians in the selection and quantification of pharmaceuticals, conducting regular discussions with stakeholders, improving communication between prescribers and dispensers, regularly supervising pharmaceutical management, and establishing an electronic pharmaceutical supply management system to monitor stock levels and expiry dates. One key informant said:

*To reduce pharmaceutical wastage, health facilities should engage in regular discussions on pharmaceutical supply management with key stakeholders and implement consistent supervision of stock management systems to ensure adherence to established standards and best practices. (P14)*

Regarding suppliers, informants recommended prioritizing the provision of pharmaceuticals based on facility needs as the primary strategy to decrease pharmaceutical waste. A key informant stated:

*To minimize pharmaceutical wastage, the EPSS should supply medicines with adequate remaining shelf life, aligned with the specific needs and consumption patterns of each facility. (P10)*

Another one added to this:

*I believe pharmaceutical wastage can only be reduced if the EPSS consistently supplies the medicines in accordance with the quantities and specifications requested. (P15)*

## 4 Discussion

Ethiopia, similar to numerous other nations worldwide, is exploring methods to decrease wastage in the pharmaceutical sector. In recent years, the FMOH has made substantial efforts to lower this wastage rate [35]. However, limited information is currently available regarding the extent of pharmaceutical wastage and its contributing factors in public health facilities within our study area. Consequently, this study seeks to determine the magnitude of pharmaceutical wastage in these specific public health facilities. It provided one of the most comprehensive mixed‐methods analyses of pharmaceutical wastage in Northwest Ethiopia. By quantifying the financial magnitude of wastage across multiple public facilities and triangulating these findings with in‐depth qualitative insights from facility CEOs and pharmacy heads, it offered a holistic understanding of both the scale and systemic drivers of wastage. This integrated approach addressed gaps in prior studies that relied solely on quantitative record reviews.

The pharmaceutical wastage rate in this study was estimated at 4.00% in terms of value, which is lower than the national figure of 8.3% [36]. Furthermore, this study recorded a lower wastage rate compared to the research conducted in 2017 in the South West Shoa Zone of the Oromia region, which reported a wastage rate of 7.5% [7]. The discrepancy may be attributed to the previous study's exclusive focus on the wastage rate of medicines, which accounted for 62% of the expired pharmaceuticals in the current study. Even a much higher wastage rate of pharmaceuticals has been found, particularly among maternal, newborn, and child health life‐saving medicines, which was 13.1%, as reported by a study conducted in public hospitals in southwest Ethiopia [37].

The findings of the current study are slightly higher than those reported in a similar study conducted in Dessie, Northeast Ethiopia, which recorded a wastage rate of 3.68% [22]. Another study conducted at public health facilities in Dire Dawa City Administration reported that the average medicine wastage rate was 3.07% between 2010 and 2012 EFY. The most wasted class of medicines was anti‐infectives, accounting for 58.3%, of which 64.6% were tablet dosage forms [17]. A study in Addis Ababa, Ethiopia, found a 2.1% wastage rate, primarily from expiration and damage. This was the highest rate among antimalarials, representing 13.1% of their total inventory value [38]. The differences between our study's findings and these previous studies might be due to variations in health facility infrastructure. The earlier studies were conducted only in major cities, likely benefiting from electronic stock management systems and regular supervision.

The average wastage rate in all selected health facilities has notably decreased. It went down from 4.72% in EFY 2012 to 4.70% in EFY 2013 and further dropped to 2.96% in EFY 2014. This reduction could be attributed to the recent implementation of IPLS in health centers and the concurrent application of both APTS and IPLS in hospitals. Yet, the figure exceeded the national target, which aimed to reduce the pharmaceutical wastage rate to below 2% by 2015 [35]. Although the wastage rate in the final study year showed improvement, it remained above this target. Whether this benchmark has been consistently achieved nationally remains unclear, suggesting that the <2% target may function more as an aspirational performance indicator rather than a consistently attained standard [17]. This underscores the need for strengthened accountability and supply chain optimization [39].

When expressed on a per‐capita basis, the estimated pharmaceutical wastage in the study zone remains lower in absolute monetary terms than figures reported in high‐income countries; however, relative to total pharmaceutical expenditure, the proportional loss is substantial. Differences in procurement scale, pricing structures, and supply chain sophistication limit direct comparability, yet the findings highlight efficiency gaps within resource‐constrained systems [22].

The annual average value of pharmaceutical waste was estimated to be 3,945,246.9 ETB in 2012 EFY, 4,612,939.81 ETB in 2013 EFY, and 3,642,932.54 ETB in 2014 EFY. This suggests that the financial impact of pharmaceutical wastage on the facilities is substantial. A Ugandan study also showed that the presence of expired pharmaceutical stock represents a significant waste of resources for nations with limited resources [40]. Health facilities must implement necessary preventive measures to minimize their financial losses.

As previously mentioned, this study identified various factors contributing to pharmaceutical waste, as perceived by healthcare professionals. Key informant interviews confirm that pharmaceutical wastage is due to several factors: not assessing needs before providing pharmaceuticals, delivering nearly expired medications to health facilities, poor stock management, inaccurate pharmaceutical quantification data, the absence of electronic stock management tools, a lack of accountability, a weak pharmaceutical monitoring system, and no system for transferring nearly expired pharmaceuticals between facilities. A study in Dire Dawa, Ethiopia, found similar results: Pharmaceutical waste is linked to several issues, such as distributing nearly expired medications to facilities, limited clinician involvement in medication selection and quantification, frequent changes in treatment protocols, and overstocked medication shelves [17].

A 2014 Ethiopian study indicated that the absence of accountability for pharmaceutical waste and the lack of tools to automatically capture it significantly contributed to the issue [41]. Similarly, the current study revealed that the lack of an electronic stock management tool, which could automatically track pharmaceutical waste in health facilities, was one of the major contributing factors.

A qualitative study conducted in South West Wollega, Ethiopia, revealed that pharmaceutical wastage was significantly influenced by poor communication between storage and dispensing units, non‐compliance with the first‐expiry, first‐out principle, and weak monitoring systems [7]. Similarly, in this study, key informants identified that inefficient inventory management systems, inadequate supervision of pharmaceutical management, and poor communication between suppliers and facilities, as well as stores and dispensing units, significantly contribute to pharmaceutical waste.

A study in West Wollega, Ethiopia, found that unsolicited bulk medicine supplies from vendors, without a facility's request, contributed to pharmaceutical wastage [2]. Similarly, key informants in this study indicated that supplying pharmaceuticals without a facility's needs or request was a significant contributor to pharmaceutical wastage.

A study conducted in Tanzania [42] revealed that overstocking, resulting from improper quantification and forecasting, was a significant factor contributing to pharmaceutical wastage. The key informants in this study also identified inappropriate quantification and forecasting as major contributors to such wastage. Additionally, both the quantitative and qualitative results of our study revealed that the delivery of nearly expired pharmaceuticals to facilities was a significant contributing factor to pharmaceutical wastage. Supporting this evidence, a study in Tanzania indicated that the short shelf life of pharmaceuticals was another key contributing factor [42].

The other important issue is that key informants in this study indicated that abrupt changes in treatment guidelines increase the wastage of pharmaceuticals. Similarly, other studies conducted in Dire Dawa and South Gondar, Ethiopia, reported that abrupt changes in treatment practices were found to be a major cause of medicine wastage in health facilities [17, 43]. A study conducted in Rwanda also revealed that abrupt changes in pharmaceutical use or treatment policies contribute to this problem [20].

The current study indicates that the scarcity of resources and increasing disposal costs are significant consequences under financial strain, as reported by key informants. In numerous developing countries, budgets for pharmaceuticals are typically limited. This limitation often results in many individuals being unable to access essential medicines. Pharmaceutical wastage further depletes the availability of these medicines to patients, thereby reducing the quality of healthcare they receive [10].

The study found that the accumulation of improperly disposed pharmaceutical waste overfills storage areas, leading to inefficient use of space in health facilities. This limits the available space for usable pharmaceutical supplies [6]. Boche et al. also noted that a lack of space was a challenge for efficient inventory management [38]. This suggests that leftover pharmaceuticals should be properly disposed of to ensure sustainable supply and use, requiring collaboration among pharmaceutical stakeholders [5].

The qualitative findings indicated that key informants recommended several strategies to decrease pharmaceutical usage. These strategies encompass enhancing inventory management, establishing better facility‐supplier and prescriber–dispenser communications, involving clinicians in drug selection and quantification, engaging in regular stakeholder discussions, conducting consistent pharmaceutical management supervision, and supplying pharmaceuticals according to facility needs. Similarly, Gebremariam et al. recommended that medicines with high wastage rates should be closely monitored and moved to the front of the shelf or reallocated to facilities with higher consumption rates to encourage the FEFO stock rotation technique [7]. A comparable study emphasized that tasks undertaken during prescribing and dispensing were deemed crucial for minimizing the pharmaceutical wastage rate [44]. A study from South Gondar, Ethiopia, also highlighted reducing drug expiry by ensuring deliveries are based on consumption patterns and involve drugs with longer shelf lives, coupled with more effective communication [45].

### 4.1 Policy and Practical Implications

The findings of the study can help develop strategies to reduce pharmaceutical waste, improve resource allocation, and ensure better patient outcomes. Key policy implications include promoting the use of generic drugs, improving inventory management, implementing expiration date policies, encouraging clinical guidelines, monitoring and evaluating waste rates, and strengthening regulatory frameworks.

Policy actions should prioritize the implementation of electronic inventory management systems, the strengthening of accountability frameworks within facilities, improved forecasting capacity building, and the establishment of structured inter‐facility redistribution mechanisms for near‐expiry medicines. Strengthened coordination between facilities and the national supplier is also critical to minimize delivery of short‐dated products.

The practical implications of the study are observable in various aspects of the healthcare system, such as drug procurement, storage, dispensing, and patient care. The key practical implications may include optimizing drug procurement, improving drug storage conditions, enhancing drug dispensing practices, promoting patient education, and collaborating with pharmaceutical companies.

### 4.2 Limitation of the Study

This study has several limitations. First, it was conducted in a single administrative zone, which may limit generalizability to other regions with different supply chain structures. Second, the cross‐sectional design captures associations and perceptions at one point in time and does not establish causality. Third, the study relied on documented wastage reports; incomplete or inconsistent recordkeeping may have led to underestimation of the true magnitude of wastage. Fourth, qualitative findings reflect participant perspectives, which may be subject to recall or social desirability bias. Fifth, it focused on wasted pharmaceuticals with price lists, excluding those registered as free. Furthermore, the findings were derived exclusively from the perspective of public health facilities, without incorporating data from health posts. However, despite these limitations, the mixed‐methods design enhances the robustness and depth of interpretation.

### 4.3 Future Research Recommendations

Future research should adopt longitudinal designs to assess causal pathways and evaluate the effectiveness of intervention strategies. Multi‐regional studies are also warranted to enhance generalizability. Additionally, economic evaluations incorporating environmental externality costs would provide a more comprehensive estimate of the true societal burden of pharmaceutical wastage.

## 5 Conclusion

The pharmaceutical wastage rate in public healthcare facilities within the study area exceeded the national target of maintaining wastage below 2% by 2015, despite a gradual reduction in the average rate over time. Expiration was identified as the leading cause of wastage. The three most significant factors contributing to this issue, as perceived by health professionals, were lack of accountability for stock‐outs and wastage at the facility level, delivery of near‐expiry pharmaceuticals with less than 6 months of remaining shelf life, and the absence of a formal system to transfer nearly expired medicines between facilities.

To mitigate pharmaceutical wastage, strengthening communication among stakeholders and implementing an electronic pharmaceutical supply management system are critical. The adoption of advanced electronic inventory management tools would provide a centralized platform for all key stakeholders, ensuring that the EPSS aligns distribution with actual facility demand and orders while prioritizing medicines with longer remaining shelf life.

Pharmaceutical wastage in the study area remains above the national benchmark, with expiry as the dominant contributor. Addressing systemic drivers—including weak accountability, forecasting limitations, and near‐expiry deliveries—requires coordinated policy and managerial reforms. Strengthening electronic stock management systems, enhancing redistribution mechanisms, and reinforcing institutional accountability are practical steps toward reducing avoidable losses and improving health system efficiency.

## Author Contributions

**Ewunetie Mekashaw Bayked**: conceived, designed, supervised, and performed the research. They also extracted, analyzed, and interpreted the data, wrote the article, and contributed to the writing and reviewing of the manuscript. **Adam Tesfaw Debeb**: conceived, designed, supervised, and performed the research. They also extracted, analyzed, and interpreted the data, wrote the article, and contributed to the writing and reviewing of the manuscript. **Getachew Moges**: conceived, designed, supervised, and performed the research. They also extracted, analyzed, and interpreted the data, wrote the article, and contributed to the writing and reviewing of the manuscript. **Husien Nurahmed Toleha**: supervised and performed the research, interpreted the data, and contributed to the writing and reviewing of the manuscript. **Teklehaimanot Fentie Wendie**: supervised and performed the research, interpreted the data, and contributed to the writing and reviewing of the manuscript. **Minimize Hassen**: supervised and performed the research, interpreted the data, and contributed to the writing and reviewing of the manuscript.

## Funding

The authors have nothing to report.

## Ethics Statement

Ethical approval was secured from the Ethical Review Committee of Research, Community Service, and Postgraduate Coordinating Office at Wollo University (ref. no. CMHS/659/14). Subsequently, this formal letter was issued to relevant parties to obtain permission to conduct the study. The study was carried out in selected public health facilities, following the acquisition of necessary permissions from the respective institutions. To maintain confidentiality and address ethical concerns, the names of these facilities were recorded and analyzed using unique identification codes.

## Consent

Informed consent was obtained from participants prior to their involvement in the study. During this process, they were informed about the purpose of the study, the reasons for their selection, their expected roles, and their right to withdraw from the study at any point. Participants were also guaranteed confidentiality, as personal identifiers were not used to collect information during the study. The study protocol was performed in accordance with the Declaration of Helsinki [46].

## Conflicts of Interest

The authors declare no conflicts of interest.

## Data Availability

The data that support the findings of this study are available within the article.
